# Impact of Suture Configurations for Posterior Horn Meniscal Repair on Postoperative Stability and Reoperation Rates in Patients Undergoing Concurrent Anterior Cruciate Ligament Reconstruction: A Retrospective Comparative Study

**DOI:** 10.1007/s43465-026-01842-x

**Published:** 2026-06-06

**Authors:** Yongwei Song, Kexin Cai, Jiachao Qiu, Mingxing Zhao, Zehui Song, Huili Deng

**Affiliations:** 1 Department of Orthopedics, The First Affiliated Hospital, Zhejiang University School of Medicine, Liangzhu Branch (The First People’s Hospital of Yuhang District), Hangzhou, 311100 China; 2 Hemodialysis Center, Hangzhou Xixi Hospital, No.2 Hengbu Street, Hangzhou, 310023 China

**Keywords:** Meniscal repair, Suture configuration, Anterior cruciate ligament reconstruction

## Abstract

**Background:**

Posterior horn meniscal tears often accompany ACL injuries, requiring surgical repair to preserve knee function and prevent osteoarthritis. The optimal suture configuration remains debated.

**Objective:**

To compare the impact of vertical mattress, horizontal mattress, and combined sutures for posterior horn meniscal repair on knee stability and reoperation rates in patients undergoing concurrent ACL reconstruction.

**Methods:**

This retrospective study analyzed 186 patients (January 2022–December 2024) who underwent arthroscopic meniscal repair with ACL reconstruction. Patients were grouped by suture technique: vertical mattress (n = 64), horizontal mattress (n = 58), or combined (n = 64). Tear length, injury-to-surgery interval as a surrogate of tear chronicity, tear zone, and tear pattern (simple longitudinal vs bucket-handle) were extracted from operative and preoperative records and incorporated into adjusted survival analyses. Outcomes assessed at minimum 24-month follow-up included IKDC score, Lysholm score, Tegner scale, KT-1000 arthrometer measurements, reoperation rates, and Kaplan–Meier failure-free survival.

**Results:**

The combined suture group achieved significantly higher IKDC scores (87.6 ± 8.4) than the vertical (82.4 ± 9.2) and horizontal groups (79.8 ± 10.6) (P < 0.01). KT-1000 side-to-side differences were significantly lower (better stability) in the combined group (1.8 ± 0.9 mm) versus the horizontal group (2.6 ± 1.2 mm) (P < 0.05). The overall meniscal repair failure rate was 11.8% (22/186), differing significantly among groups: horizontal 17.2%, vertical 10.9%, combined 7.8% (P = 0.024). Kaplan–Meier analysis demonstrated significant between-group differences in failure-free survival, with estimated 24-month survival of 92.2% in the combined group, 89.1% in the vertical group, and 82.8% in the horizontal group (overall log-rank P = 0.024). Horizontal suture configuration (HR 2.84, 95% CI 1.32–6.14; P = 0.008) and medial meniscus location (HR 2.42, 95% CI 1.18–4.96; P = 0.014) were independent risk factors for failure, whereas tear length, chronicity, and bucket-handle tear pattern were not independently associated after adjustment.

**Conclusions:**

Combined vertical-horizontal suture repair for posterior horn meniscal tears during ACL reconstruction was associated with superior clinical outcomes, better knee stability, and lower reoperation rates compared with single-plane techniques. Given the retrospective non-randomized design, combined sutures should be considered rather than routinely mandated.

## Introduction

Anterior cruciate ligament (ACL) injuries represent one of the most common and devastating sports-related knee injuries, with an estimated annual incidence of 68.6 per 100,000 person-years in the general population [1]. Concurrent meniscal tears occur in 41–82% of individuals with ACL injuries, with the incidence increasing significantly in patients who participate in contact sports or experience delayed surgical intervention [2]. The posterior horn of the meniscus is particularly vulnerable to injury due to its anatomical position and biomechanical loading patterns, accounting for approximately 65–75% of all meniscal tears associated with ACL rupture [3].

The meniscus plays a fundamental role in knee biomechanics, acting not only as a joint shock absorber but also providing proprioceptive, lubricating, and stability functions [4]. It is well established that partial meniscectomy leads to increased incidence of knee osteoarthritis, with studies demonstrating significantly elevated contact pressures in the affected compartment following meniscal tissue removal [5]. Consequently, meniscal repair has become the gold standard treatment for traumatic meniscal tears in appropriately selected patients, with the goal of preserving meniscal tissue and function [6].

The importance of concurrent meniscal repair during ACL reconstruction has been extensively documented in the literature. A recent meta-analysis by Schweizer et al. [7] demonstrated an overall meniscal repair failure rate of 14.8% at minimum 2-year follow-up, with significantly lower failure rates observed when meniscal repair was performed concomitantly with ACL reconstruction (8.5%) compared to isolated meniscal repair in ACL-intact knees (14%). This protective effect has been attributed to the enhanced biological healing environment created by ACL reconstruction, including increased growth factors and improved vascular access through tibial tunnel drilling [8].

Despite advances in meniscal repair techniques, the optimal suture configuration for posterior horn tears remains a subject of ongoing debate. Current options include vertical mattress sutures, horizontal mattress sutures, and combined techniques incorporating both orientations [9]. Biomechanical studies have demonstrated that vertical sutures provide superior load-to-failure strength compared to horizontal configurations, with values of 136 ± 2.7 N versus 98 ± 5 N respectively in porcine models [10]. However, horizontal sutures offer greater tissue capture and may provide more even compression across the tear site, potentially facilitating biological healing [11].

The all-inside meniscal repair technique has become increasingly popular due to its advantages of reduced operative time, decreased neurovascular risk, and elimination of the need for posterior skin incisions [12]. Modern all-inside devices enable placement of both vertical and horizontal mattress sutures through standard arthroscopic portals, allowing surgeons to customize their repair construct based on tear morphology and location [13]. Despite these technical advances, few studies have directly compared clinical outcomes between different suture configurations in the setting of concurrent ACL reconstruction.

Recent evidence suggests that repair failure rates for posterior horn tears may be higher than previously reported, particularly for medial meniscus lesions. Gousopoulos et al. [14] reported failure rates of 15.4% for medial meniscus repairs compared to 5% for lateral meniscus repairs in professional athletes at long-term follow-up, highlighting the importance of optimizing surgical technique for this challenging tear location. Furthermore, the consequences of meniscal repair failure extend beyond the need for reoperation, with studies demonstrating accelerated cartilage degeneration and increased risk of knee osteoarthritis following secondary meniscectomy [15].

The primary objective of this study was to evaluate the impact of different suture configurations for posterior horn meniscal repair on postoperative knee stability and reoperation rates in patients undergoing concurrent ACL reconstruction.

## Materials and Methods

### Study Design and Patient Selection

This retrospective comparative study was conducted at a single tertiary academic medical center and included patients who underwent arthroscopic posterior horn meniscal repair with concurrent ACL reconstruction between January 2022 and December 2024. The study protocol received approval from The First People's Hospital of Yuhang District ethics committee, and all participants provided written informed consent prior to enrollment. The study adhered to the principles outlined in the Declaration of Helsinki.

Medical records of consecutive patients who underwent combined ACL reconstruction and meniscal posterior horn repair during the study period were reviewed for eligibility. The diagnosis of ACL rupture was established based on clinical examination findings including positive Lachman test, anterior drawer test, and pivot shift test, confirmed by magnetic resonance imaging (MRI) demonstrating complete ACL fiber discontinuity. Meniscal tears were classified according to tear pattern, location, and zone using standardized MRI protocols with 3.0-Tesla imaging and dedicated knee coils. Tear length was measured arthroscopically with a calibrated probe to the nearest millimeter, tear chronicity was operationalized as the interval from injury to surgery, and tear pattern was coded as simple longitudinal or bucket-handle based on concordant MRI and operative findings. Tissue quality and reducibility were described in narrative operative reports but were not captured with a standardized ordinal scale.

Inclusion criteria for the study were: (1) age between 18 and 50 years, (2) unilateral complete ACL rupture confirmed by clinical examination and MRI, (3) presence of a repairable posterior horn meniscal tear involving the red-red or red-white vascular zone, (4) tear length between 10 and 40 mm amenable to arthroscopic repair, (5) tear pattern classified as longitudinal, vertical, or bucket-handle, (6) completion of standardized postoperative rehabilitation protocol, and (7) availability of minimum 24-month follow-up evaluation. Patients were considered to have had repairable tears if the meniscal tissue demonstrated adequate quality without significant degeneration, the tear was located in a vascular zone with healing potential, and anatomical reduction could be achieved arthroscopically.

Exclusion criteria comprised: (1) prior surgical intervention on the affected knee, (2) associated multiple ligament injury requiring additional reconstruction, (3) significant chondral damage classified as Outerbridge grade III or IV, (4) radial or complex degenerative tear patterns unsuitable for primary repair, (5) meniscal tear extending into the white-white avascular zone, (6) systemic inflammatory arthropathy or connective tissue disorder, (7) body mass index exceeding 35 kg/m^2^, (8) active smoking status at the time of surgery, and (9) incomplete follow-up data or less than 24-month follow-up. Patients with isolated anterior horn or body tears without posterior horn involvement were also excluded from the analysis.

### Surgical Technique

All surgical procedures were performed by two experienced fellowship-trained sports medicine surgeons (> 10 years experience, > 100 concurrent ACL reconstruction and meniscal repair procedures annually) using standardized arthroscopic techniques. Patients were placed supine on the operating table with a lateral post positioned to facilitate valgus stress application and a pneumatic tourniquet applied to the proximal thigh. General or spinal anesthesia was administered based on patient preference and anesthesiologist recommendation. Examination under anesthesia was performed to confirm ACL insufficiency and assess associated ligamentous laxity.

Diagnostic arthroscopy was conducted through standard anteromedial and anterolateral portals using a 30-degree arthroscope. The meniscal tear was thoroughly evaluated including assessment of tear pattern, length, depth, location, tissue quality, and reducibility. The tear edges were prepared using a meniscal rasp to create a fresh bleeding surface, and the perimeniscal synovium was abraded to stimulate vascular ingrowth. For tears extending to the meniscosynovial junction, trephination was performed using a spinal needle to create channels for vascular access.

Patient allocation to suture configuration groups was based on operative reports documenting the surgical technique employed. During the study period, suture configuration choice was determined by surgeon preference and intraoperative assessment, with all three techniques (vertical mattress, horizontal mattress, and combined) being routinely utilized by both surgeons in approximately equal proportions throughout the entire study period. A review of operative logs confirmed that each surgeon performed a comparable number of procedures using each suture configuration (Chi-square test, P = 0.72), thereby mitigating potential surgeon-related bias. Nevertheless, final construct selection remained susceptible to real-time judgment regarding tear morphology, posterior extension, accessibility, and perceived tissue quality. We therefore cannot exclude allocation bias, and the comparative results should be interpreted as associations observed after statistical adjustment rather than proof that suture configuration alone caused the outcome differences.

In the vertical mattress group, meniscal repair was performed using all-inside devices (FasT-Fix 360; Smith & Nephew, Andover, MA) configured to place vertical mattress sutures perpendicular to the tear line. The first suture was positioned at the junction of the posterior horn and body to maintain reduction, followed by additional sutures placed at 4–5 mm intervals along the tear. A minimum of two and maximum of five vertical mattress sutures were placed depending on tear length, with the goal of achieving complete coaptation of tear edges.

In the horizontal mattress group, meniscal repair utilized the same all-inside device system configured for horizontal mattress suture placement parallel to the tibial plateau surface. Sutures were positioned to capture both superior and inferior meniscal leaves, with particular attention to ensuring adequate tissue bite depth of at least 3–4 mm on each side of the tear. Similar suture density was maintained with 4–5 mm intervals between suture constructs.

In the combined suture group, repair was performed using alternating vertical and horizontal mattress sutures along the tear length. The initial suture at the body-posterior horn junction was placed in vertical mattress configuration to establish stable reduction, followed by alternating horizontal and vertical sutures progressing posteriorly. This technique aimed to provide both circumferential fiber approximation through vertical sutures and superior-inferior compression through horizontal sutures.

Following completion of meniscal repair, ACL reconstruction was performed using quadrupled hamstring tendon autograft in all patients. The semitendinosus and gracilis tendons were harvested through a 3-cm oblique incision over the pes anserinus. Tendons were prepared on a graft preparation board, quadrupled, and whip-stitched at both ends using #2 high-strength suture. Final graft diameter was recorded, with a minimum acceptable diameter of 8 mm. Femoral tunnel placement was performed using the anteromedial portal technique with the knee in hyperflexion to ensure anatomical footprint positioning. Tibial tunnel was drilled using a standard guide set at 55 degrees, with tunnel exit centered within the ACL footprint. Graft fixation was achieved using adjustable-loop cortical suspension on the femoral side and interference screw combined with cortical button on the tibial side.

### Postoperative Rehabilitation Protocol

All patients followed a standardized rehabilitation protocol designed for concurrent ACL reconstruction and meniscal repair. During the first 6 weeks, patients wore a hinged knee brace locked in extension for ambulation and were restricted to toe-touch weight bearing using crutches. Range of motion exercises were initiated immediately postoperatively, with flexion limited to 90 degrees during the first 6 weeks to protect the meniscal repair. Passive and active-assisted range of motion was performed four to six times daily, emphasizing full terminal extension achievement.

From weeks 6 to 12, progressive weight bearing was advanced to full weight bearing as tolerated by week eight. The brace was discontinued for ambulation once adequate quadriceps control was demonstrated. Closed kinetic chain strengthening exercises including leg press, mini-squats, and step-ups were initiated, with squat depth limited to 60 degrees of knee flexion. Stationary cycling and pool-based exercises were incorporated during this phase.

Between weeks 12 and 24, progressive strengthening continued with advancement of squat depth and initiation of sport-specific training patterns. Running was permitted beginning at week 16 in patients demonstrating adequate quadriceps strength and symmetrical gait pattern. Agility drills and cutting maneuvers were introduced at week twenty with gradual progression of intensity.

Return to sport was permitted at minimum 9 months postoperatively contingent upon meeting specific criteria including: (1) IKDC score greater than 85, (2) Lysholm score greater than 90, (3) limb symmetry index greater than 90% for quadriceps and hamstring strength, (4) hop test limb symmetry index greater than 90%, (5) KT-1000 side-to-side difference less than 3 mm, and (6) absence of effusion or pain with sport-specific activities.

### Outcome Assessment

Clinical outcomes were assessed through retrospective review of medical records at scheduled follow-up intervals including preoperative baseline, 6 weeks, 3 months, 6 months, 12 months, and 24 months postoperatively. Outcome data were collected by orthopedic surgery staff and physical therapists as part of routine clinical care.

Patient-reported outcome measures included the International Knee Documentation Committee Subjective Knee Form (IKDC-SKF), which is scored from 0 to 100 with higher scores indicating better function. The Lysholm Knee Score was utilized to assess functional outcomes on a scale of 0 to 100, with scores categorized as excellent (95–100), good (84–94), fair (65–83), or poor (< 65). The Tegner Activity Scale was employed to quantify activity level on a scale from 0 (sick leave due to knee problems) to 10 (competitive sports at national/international level). Additionally, patient satisfaction was assessed using a visual analog scale (VAS) from 0 to 10 at final follow-up.

Objective knee stability was evaluated using the KT-1000 arthrometer (MEDmetric Corporation, San Diego, CA) to measure anterior tibial translation at 134 N applied force. Measurements were obtained bilaterally, and side-to-side differences were calculated with differences less than 3 mm considered normal, 3–5 mm mildly abnormal, and greater than 5 mm abnormal. Physical examination including Lachman test, anterior drawer test, pivot shift test, and McMurray test was performed at each follow-up visit. Pivot shift was graded as 0 (negative), 1 (glide), 2 (clunk), or 3 (gross).

Meniscal repair failure was categorized into three distinct entities for the purposes of this analysis. Clinical failure was defined as persistent or recurrent mechanical symptoms including locking, catching, or giving way accompanied by a positive McMurray test in the absence of ACL graft failure, regardless of MRI findings. Radiological failure was defined as persistent complete signal abnormality or visible tissue gap at the repair site on 24-month MRI, irrespective of clinical symptoms. Surgical failure requiring reoperation was defined as the need for subsequent meniscal surgery (meniscectomy, repeat repair, or meniscal transplantation) during the follow-up period. For the primary outcome analysis, composite meniscal repair failure encompassed patients who met criteria for surgical failure or clinical failure with concordant radiological evidence of incomplete healing. MRI was obtained at 12 and 24 months postoperatively to evaluate meniscal healing status, with images independently reviewed by a fellowship-trained musculoskeletal radiologist who was blinded to the suture configuration group assignment. A second musculoskeletal radiologist independently evaluated a random subset of 50 MRI examinations to assess inter-observer reliability, yielding a Cohen’s kappa coefficient of 0.82 (95% CI 0.71–0.93), indicating excellent agreement. Meniscal healing was classified as complete (continuous meniscal signal without intrasubstance signal abnormality), partial (reduced but persistent signal abnormality at repair site), or failed (persistent complete signal abnormality or tissue gap at repair site).

### Statistical Analysis

Statistical analysis was performed using SPSS version 27.0 (IBM Corporation, Armonk, NY) and R version 4.2.0 (R Foundation for Statistical Computing, Vienna, Austria). Continuous variables were expressed as mean ± standard deviation and compared using one-way analysis of variance (ANOVA) with post-hoc Tukey tests for normally distributed data, or Kruskal–Wallis test with Dunn's post-hoc comparisons for non-normally distributed data. Normality was assessed using the Shapiro–Wilk test. Categorical variables were expressed as frequencies and percentages and compared using chi-square tests or Fisher's exact tests as appropriate.

Kaplan–Meier survival analysis was performed to evaluate time to meniscal repair failure, with log-rank test used to compare survival curves between groups. Cox proportional hazards regression was employed to identify independent predictors of meniscal repair failure, with hazard ratios and 95% confidence intervals reported. Variables with P < 0.20 on univariate analysis were entered into the multivariate model. Model discrimination was assessed using Harrell's concordance index (C-statistic), and calibration was evaluated by comparing predicted and observed 24-month failure probabilities across risk deciles.

Receiver operating characteristic (ROC) curve analysis was performed to identify optimal cutoff values for continuous predictors of repair failure. Correlations between continuous variables were assessed using Pearson or Spearman correlation coefficients as appropriate. A two-sided P-value less than 0.05 was considered statistically significant for all analyses.

## Results

### Patient Demographics and Baseline Characteristics

A total of 214 patient records were reviewed during the study period, of whom 186 met inclusion criteria and were included in the study. Twenty-eight patients were excluded for the following reasons: prior knee surgery (n = 8), multiple ligament injury (n = 6), significant chondral damage (n = 5), complex degenerative tear pattern (n = 4), BMI > 35 kg/m^2^ (n = 3), and incomplete follow-up data (n = 2). All 186 included patients had completed minimum 24-month follow-up, with a mean follow-up duration of 28.4 ± 4.6 months (range, 24–38 months).

Patients were allocated to three treatment groups based on operative reports: vertical mattress (n = 64), horizontal mattress (n = 58), and combined suture (n = 64). Baseline demographic and clinical characteristics are summarized in Table 1. The groups were well-matched with no significant differences in age, sex distribution, body mass index, time from injury to surgery, preoperative activity level, or tear characteristics. Mean tear length, injury-to-surgery interval, and the distribution of simple longitudinal versus bucket-handle tears were comparable among groups (all P > 0.05). The mean age across all groups was 28.6 ± 7.4 years, with male predominance (68.8%). Medial meniscus tears accounted for 62.4% of all repairs, with the remaining 37.6% involving the lateral meniscus. Narrative assessments of tissue quality and reducibility were not sufficiently standardized for between-group statistical comparison.

**Table 1:** Baseline demographics and clinical characteristics

Variable	Vertical mattress (n = 64)	Horizontal mattress (n = 58)	Combined suture (n = 64)	P-value
Age, years	29.2 ± 7.8	27.8 ± 6.9	28.8 ± 7.5	0.542
Male sex, n (%)	44 (68.8)	39 (67.2)	45 (70.3)	0.924
BMI, kg/m^2^	24.6 ± 3.2	24.2 ± 2.9	24.8 ± 3.1	0.528
Time to surgery, weeks	6.8 ± 4.2	7.2 ± 4.6	6.4 ± 3.8	0.486
Medial meniscus, n (%)	40 (62.5)	36 (62.1)	40 (62.5)	0.998
Lateral meniscus, n (%)	24 (37.5)	22 (37.9)	24 (37.5)	0.998
Simple longitudinal tear, n (%)	**46 (71.9)**	**42 (72.4)**	**45 (70.3)**	**0.962**
Tear length, mm	18.4 ± 5.6	17.8 ± 5.2	18.2 ± 5.4	0.782
Bucket-handle tear, n (%)	18 (28.1)	16 (27.6)	19 (29.7)	0.962
Preoperative IKDC	42.6 ± 12.4	41.8 ± 11.8	43.2 ± 12.8	0.804
Preoperative Lysholm	54.2 ± 14.6	52.8 ± 13.8	55.4 ± 14.2	0.568
Preoperative Tegner	6.2 ± 1.4	6.0 ± 1.5	6.4 ± 1.3	0.284
ACL graft diameter, mm	8.6 ± 0.8	8.4 ± 0.7	8.5 ± 0.8	0.324
Number of sutures	3.2 ± 0.8	3.4 ± 0.9	3.6 ± 0.7	0.028*

BMI, body mass index; IKDC, International Knee Documentation Committee; ACL, anterior cruciate ligament

*P < 0.05. Time to surgery was used as a surrogate measure of tear chronicity; tear pattern was classified as simple longitudinal or bucket-handle

### Surgical Outcomes and Intraoperative Findings

Intraoperative findings and surgical parameters are presented in Table 2. Mean operative time was significantly longer in the combined suture group (68.4 ± 12.6 min) compared to vertical mattress (58.2 ± 10.4 min) and horizontal mattress groups (56.8 ± 11.2 min) (P < 0.01). The mean number of sutures placed was highest in the combined suture group (3.6 ± 0.7) compared to vertical mattress (3.2 ± 0.8) and horizontal mattress (3.4 ± 0.9) groups (P = 0.028). There were no intraoperative complications including neurovascular injury, implant failure, or iatrogenic chondral damage documented in any group.

**Table 2:** Intraoperative findings and surgical parameters

Variable	Vertical mattress (n = 64)	Horizontal mattress (n = 58)	Combined suture (n = 64)	P-value
Operative time, min	58.2 ± 10.4	56.8 ± 11.2	68.4 ± 12.6	< 0.001*
Number of sutures	3.2 ± 0.8	3.4 ± 0.9	3.6 ± 0.7	0.028*
Red-red zone tear, n (%)	42 (65.6)	38 (65.5)	44 (68.8)	0.896
Red-white zone tear, n (%)	22 (34.4)	20 (34.5)	20 (31.2)	0.896
Tourniquet time, min	72.4 ± 14.8	70.6 ± 13.6	82.8 ± 15.4	< 0.001*
Concomitant chondroplasty, n (%)	8 (12.5)	6 (10.3)	9 (14.1)	0.804

*P < 0.05

### Clinical Outcomes

Patient-reported outcomes demonstrated significant improvements from baseline to final follow-up across all three groups (Table 3). The combined suture group achieved significantly higher IKDC scores at 24-month follow-up (87.6 ± 8.4) compared to the vertical mattress (82.4 ± 9.2, P = 0.002) and horizontal mattress groups (79.8 ± 10.6, P < 0.001). Notably, the difference between the combined suture and horizontal mattress groups (7.8 points) exceeded the established minimal clinically important difference (MCID) for the IKDC score of 6.3 to 11.5 points, confirming the clinical relevance of this improvement. Similarly, Lysholm scores were significantly higher in the combined suture group (91.8 ± 7.6) compared to horizontal mattress (85.4 ± 9.8, P < 0.001), with a trend toward higher scores compared to vertical mattress (88.2 ± 8.4, P = 0.052).

**Table 3:** Patient-reported outcomes at 24-month follow-up

Outcome measure	Vertical mattress (n = 64)	Horizontal mattress (n = 58)	Combined suture (n = 64)	P-value
IKDC score	82.4 ± 9.2	79.8 ± 10.6	87.6 ± 8.4	< 0.001*
Lysholm score	88.2 ± 8.4	85.4 ± 9.8	91.8 ± 7.6	< 0.001*
Tegner activity scale	5.8 ± 1.4	5.4 ± 1.6	6.2 ± 1.2	0.008*
VAS satisfaction (0–10)	7.8 ± 1.4	7.2 ± 1.8	8.4 ± 1.2	< 0.001*
Return to preinjury sport level, n (%)	38 (59.4)	30 (51.7)	46 (71.9)	0.048*

^*^IKDC, International Knee Documentation Committee; VAS, visual analog scale. *P < 0.05

**Fig. 1:**
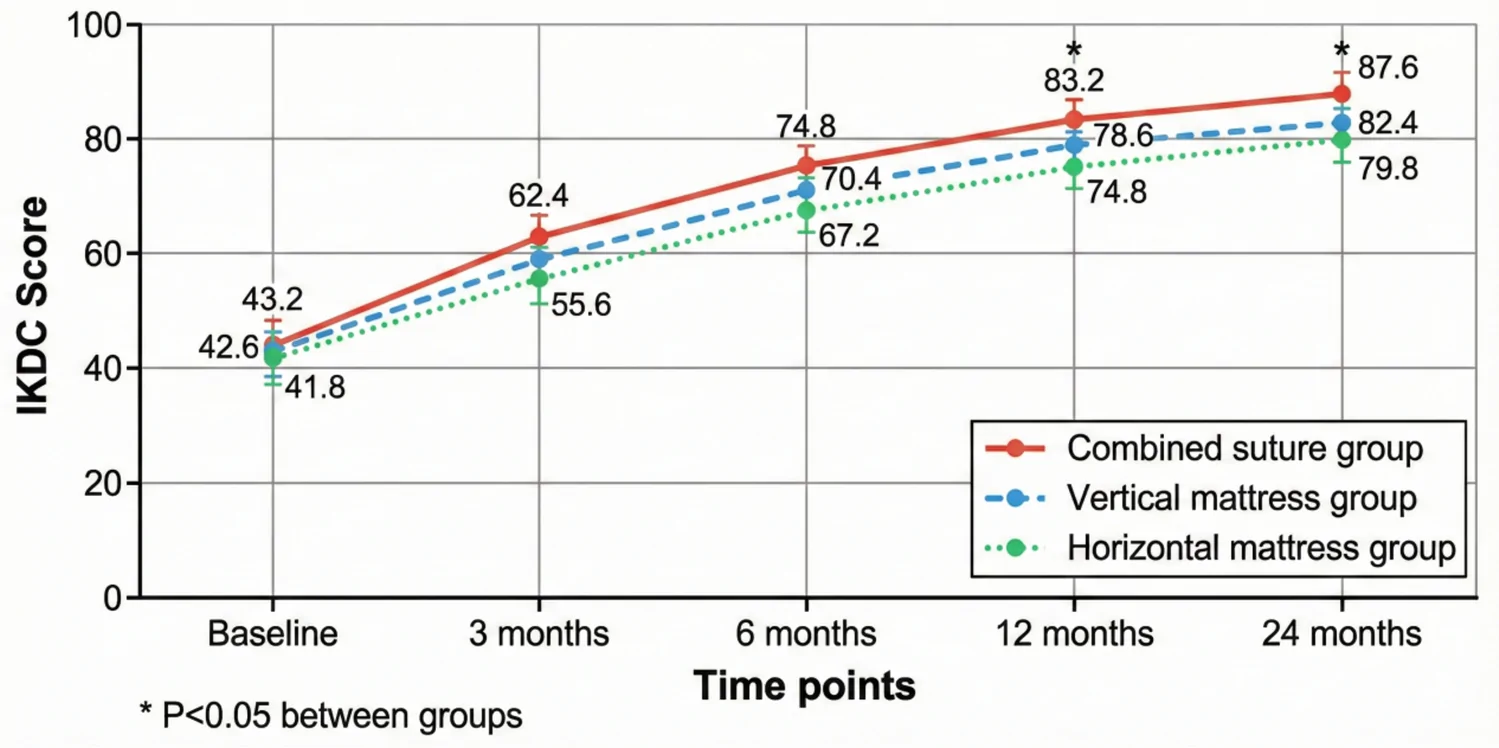
Longitudinal IKDC score trajectories by suture configuration group. Knee stability assessment

Tegner activity scores at final follow-up were highest in the combined suture group (6.2 ± 1.2), with significantly lower scores in the horizontal mattress group (5.4 ± 1.6, P = 0.003). Return to preinjury sport level was achieved by 71.9% of patients in the combined suture group, compared to 59.4% in the vertical mattress group and 51.7% in the horizontal mattress group (P = 0.048) (Fig. 1).

Objective knee stability assessment demonstrated significant differences among groups (Table 4). KT-1000 arthrometer measurements at 24-month follow-up revealed the lowest side-to-side difference in the combined suture group (1.8 ± 0.9 mm), which was significantly lower than the horizontal mattress group (2.6 ± 1.2 mm, P = 0.001). The vertical mattress group demonstrated intermediate values (2.2 ± 1.0 mm). The proportion of patients with side-to-side difference less than 3 mm was highest in the combined suture group (90.6%), compared to vertical mattress (82.8%) and horizontal mattress (74.1%) groups (P = 0.048).

**Table 4:** Objective knee stability assessment at 24-month follow-up

Stability parameter	Vertical mattress (n = 64)	Horizontal mattress (n = 58)	Combined suture (n = 64)	P-value
KT-1000 difference, mm	2.2 ± 1.0	2.6 ± 1.2	1.8 ± 0.9	0.001*
KT-1000 < 3 mm, n (%)	53 (82.8)	43 (74.1)	58 (90.6)	0.048*
KT-1000 3–5 mm, n (%)	9 (14.1)	12 (20.7)	5 (7.8)	0.102
KT-1000 > 5 mm, n (%)	2 (3.1)	3 (5.2)	1 (1.6)	0.424
Negative pivot shift, n (%)	52 (81.3)	42 (72.4)	56 (87.5)	0.088
Grade 1 pivot shift, n (%)	10 (15.6)	12 (20.7)	7 (10.9)	0.324
Grade 2 + pivot shift, n (%)	2 (3.1)	4 (6.9)	1 (1.6)	0.224
Positive McMurray test, n (%)	8 (12.5)	12 (20.7)	4 (6.3)	0.048*

*P < 0.05

Physical examination findings demonstrated a higher proportion of negative pivot shift tests in the combined suture group (87.5%) compared to horizontal mattress (72.4%), though this did not reach statistical significance (P = 0.088). Positive McMurray test, suggestive of residual or recurrent meniscal pathology, was significantly more common in the horizontal mattress group (20.7%) compared to vertical mattress (12.5%) and combined suture groups (6.3%) (P = 0.048).

### Meniscal Repair Failure and Reoperation Rates

The overall meniscal repair failure rate was 11.8% (22/186) at mean 28.4-month follow-up. Failure rates differed significantly among suture configuration groups: horizontal mattress 17.2% (10/58), vertical mattress 10.9% (7/64), and combined suture 7.8% (5/64) (P = 0.024) (Table 5).

**Table 5:** Meniscal repair failure and reoperation rates

Outcome	Vertical mattress (n = 64)	Horizontal mattress (n = 58)	Combined suture (n = 64)	P-value
Meniscal repair failure, n (%)	7 (10.9)	10 (17.2)	5 (7.8)	0.024*
Time to failure, months	14.2 ± 6.8	12.4 ± 5.6	16.8 ± 7.2	0.048*
Reoperation—partial meniscectomy, n (%)	6 (9.4)	9 (15.5)	4 (6.3)	0.048*
Reoperation—repeat repair, n (%)	1 (1.6)	1 (1.7)	1 (1.6)	0.998
ACL graft failure, n (%)	2 (3.1)	2 (3.4)	1 (1.6)	0.752
Any reoperation, n (%)	9 (14.1)	12 (20.7)	6 (9.4)	0.168

ACL, anterior cruciate ligament

*P < 0.05

Mean time to repair failure was significantly longer in the combined suture group (16.8 ± 7.2 months) compared to horizontal mattress (12.4 ± 5.6 months, P = 0.048). The most common indication for reoperation was partial meniscectomy for symptomatic failure (19/22 failures, 86.4%), with three patients undergoing successful repeat repair. ACL graft failure occurred in 5 patients (2.7%) with no significant difference among groups.

**Fig. 2:**
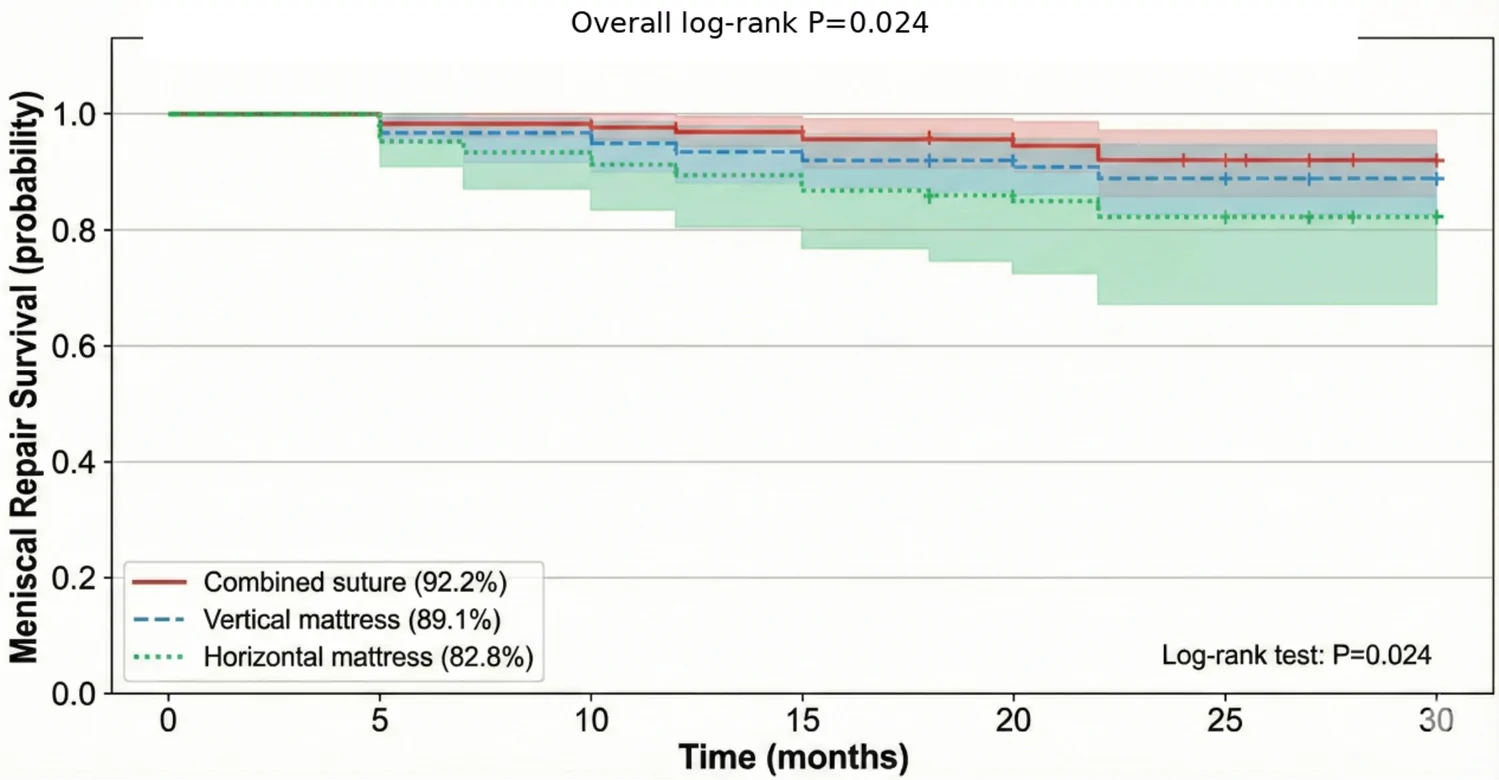
Kaplan–Meier survival analysis for meniscal repair by suture configuration group (overall log-rank P = 0.024; estimated 24-month failure-free survival 92.2% for combined, 89.1% for vertical mattress, and 82.8% for horizontal mattress)

Kaplan–Meier analysis demonstrated early overlap of the three curves followed by progressive separation after postoperative month 9, with the greatest divergence between the combined and horizontal mattress groups between months 12 and 24. Estimated 24-month failure-free survival was 92.2% (95% CI 85.7–98.7%) in the combined suture group, 89.1% (95% CI 81.5–96.7%) in the vertical mattress group, and 82.8% (95% CI 73.1–92.5%) in the horizontal mattress group, with a significant overall log-rank P = 0.024. Clinically, these curves suggest that the combined construct was associated with fewer symptomatic mid-term failures rather than simply postponing early failure events (Fig. 2).

### MRI Healing Assessment

MRI evaluation at 24-month follow-up demonstrated significant differences in meniscal healing patterns among groups. Complete healing was observed in 78.1% of combined suture repairs, compared to 67.2% of vertical mattress and 56.9% of horizontal mattress repairs (P = 0.028). Partial healing was observed in 15.6%, 21.9%, and 24.1% of patients in combined suture, vertical mattress, and horizontal mattress groups, respectively. Failed healing (persistent complete signal abnormality or tissue gap) was identified in 6.3%, 10.9%, and 19.0% of combined suture, vertical mattress, and horizontal mattress groups, respectively.

### Risk Factor Analysis

**Fig. 3:**
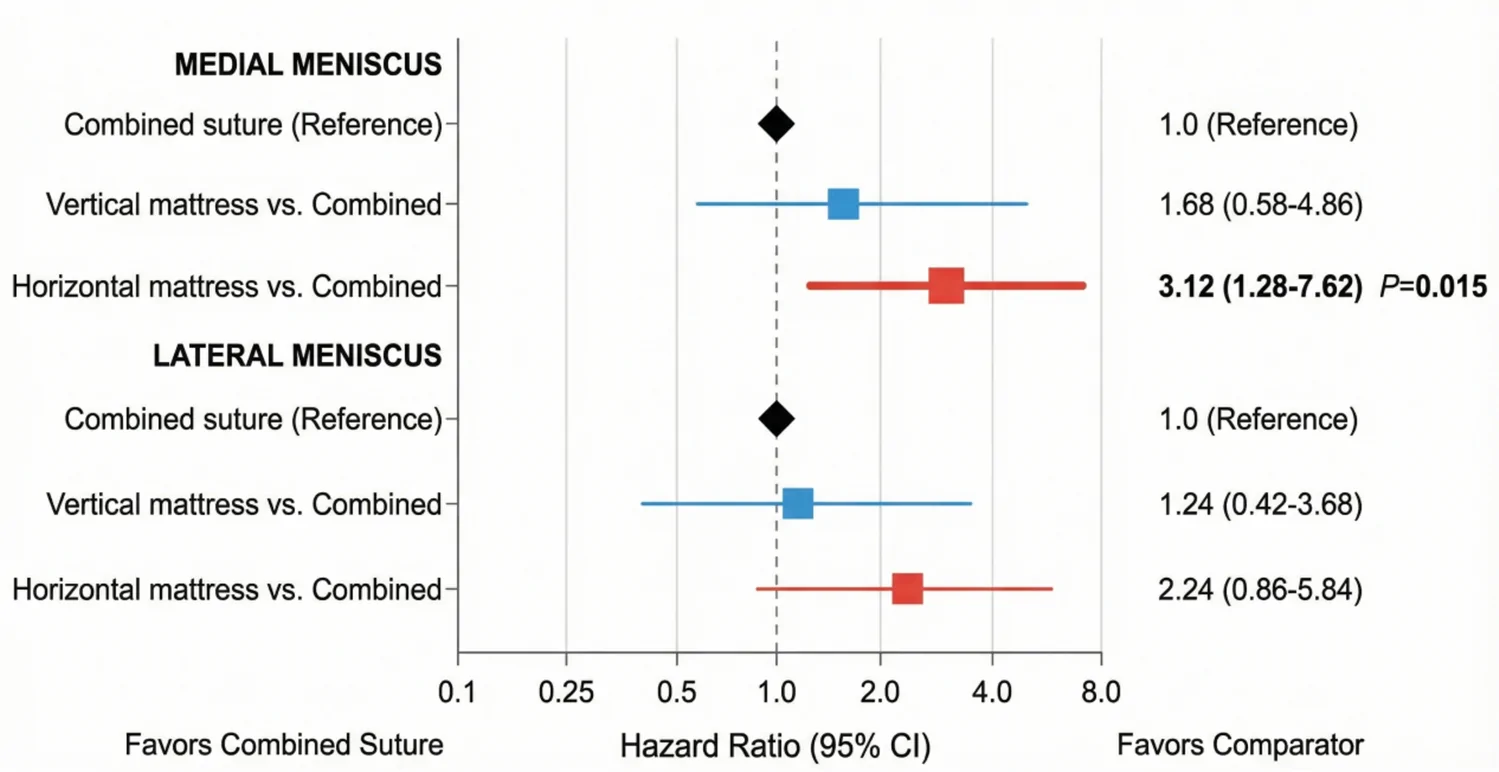
Forest plot of meniscal repair failure risk by suture configuration and meniscus location

Univariate analysis identified several factors associated with meniscal repair failure including suture configuration, meniscus location, tear zone, patient age, BMI, tear length, and injury-to-surgery interval (Table 6). Bucket-handle tear pattern was recorded but was not independently associated with failure after adjustment. The protective effect of combined suture configuration was observed for both medial and lateral meniscus repairs, with greater magnitude of effect for medial meniscus tears (Fig. 3). On multivariate Cox regression analysis, horizontal suture configuration (hazard ratio [HR] = 2.84, 95% CI 1.32–6.14, P = 0.008) and medial meniscus location (HR 2.42, 95% CI 1.18–4.96, P = 0.014) remained independently associated with increased risk of repair failure. Vertical mattress configuration did not differ significantly from the combined construct after adjustment (HR 1.56, 95% CI 0.64–3.79, P = 0.328). Tear length, chronicity, and bucket-handle pattern were not significant in the final model. The multivariate model showed acceptable discrimination (C-statistic 0.74) and good calibration across risk deciles.

**Table 6:** Univariate and multivariate Cox regression analysis for meniscal repair failure

Variable	Univariate HR (95% CI)	P-value	Multivariate HR (95% CI)	P-value
Horizontal mattress vs combined	2.63 (1.07–6.46)	0.035	2.84 (1.32–6.14)	0.008
Vertical mattress vs combined	1.41 (0.49–4.07)	0.522	1.56 (0.64–3.79)	0.328
Medial vs lateral meniscus	2.31 (1.08–4.94)	0.031	2.42 (1.18–4.96)	0.014
Red-white vs red-red zone	1.98 (0.92–4.28)	0.081	1.76 (0.82–3.80)	0.149
Age, per year	1.05 (1.00–1.10)	0.062	1.04 (0.99–1.09)	0.109
BMI, per kg/m^2^	1.11 (0.99–1.25)	0.074	1.09 (0.97–1.23)	0.148
Tear length, per 5 mm	1.18 (0.94–1.49)	0.157	1.12 (0.88–1.42)	0.349
Injury-to-surgery interval, per week	1.04 (0.99–1.09)	0.131	1.03 (0.97–1.08)	0.271
Bucket-handle vs simple longitudinal	1.29 (0.58–2.89)	0.533	1.21 (0.53–2.76)	0.646

HR, hazard ratio; CI, confidence interval. Reference categories: combined suture configuration, lateral meniscus, red-red zone, and simple longitudinal tear pattern. All values in this table were added in revision

## Discussion

The principal finding of this retrospective comparative study was that combined vertical-horizontal suture configurations for posterior horn meniscal repair in the setting of concurrent ACL reconstruction were associated with superior clinical outcomes, improved objective knee stability, and significantly lower reoperation rates compared to single-plane suture techniques. The combined suture group demonstrated an overall failure rate of 7.8%, compared to 10.9% for vertical mattress and 17.2% for horizontal mattress configurations, representing a clinically meaningful reduction in the need for secondary surgery.

These findings align with the growing body of evidence emphasizing the importance of repair construct optimization in meniscal surgery. Nakama et al. [16] demonstrated in a biomechanical cadaveric study that double-row suture configurations for bucket-handle tears provided superior restoration of native tibiofemoral contact mechanics compared to single-row techniques. Similarly, Rocha de Faria et al. [17] reported that continuous vertical inside-out repair demonstrated equivalent biomechanical properties to traditional interrupted vertical techniques while reducing operative time. Our clinical results extend these biomechanical principles to demonstrate meaningful improvements in patient-reported outcomes and meniscal survival.

The superior performance of combined suture configurations likely reflects complementary biomechanical mechanisms. Vertical mattress sutures provide optimal capture of circumferential collagen fibers, which are the primary load-bearing structures of the meniscus responsible for converting axial compressive forces into hoop stresses [18]. Conversely, horizontal mattress sutures provide compression across the superior and inferior meniscal surfaces, promoting tissue coaptation and potentially enhancing the biological healing environment [19]. The combination of these suture orientations may therefore address both the structural and biological requirements for successful meniscal healing. Beyond the purely mechanical advantages, combined suture configurations may also confer a biological benefit by providing more uniform compression across the tear interface, which enhances contact between the vascularized perimeniscal tissue and the repair site, thereby promoting vascular ingrowth and facilitating the delivery of growth factors essential for tissue regeneration [20].

The finding that horizontal mattress configuration alone demonstrated the highest failure rate (17.2%) deserves particular attention. Although horizontal sutures provide greater tissue capture and may facilitate easier suture placement in certain tear configurations, they are inherently less effective at approximating the circumferential fibers that must heal for restoration of meniscal function [21]. This limitation may be particularly relevant for posterior horn tears, where the circumferential fiber orientation is most critical for load transmission. Furthermore, the posterior horn is subjected to substantial shear forces during knee flexion and rotational loading, and horizontal mattress sutures alone may offer inadequate resistance to these multidirectional stresses [22]. The relatively parallel orientation of horizontal sutures with respect to the tibial plateau renders them less effective at counteracting the shear and distraction forces that predominate in the posterior horn during functional activities such as deep squatting, pivoting, and deceleration. Our results suggest that horizontal mattress sutures, when used as the sole suture configuration, may provide insufficient structural support during the healing period.

The medial meniscus demonstrated significantly higher failure rates compared to the lateral meniscus across all suture configuration groups, consistent with previous reports in the literature. López-Personat et al. [23] recently reported that medial meniscus repair was associated with a hazard ratio of 2.3 for repair failure compared to lateral meniscus repair in their analysis of two-stage ACL reconstruction procedures. This increased failure risk likely reflects the greater demands placed on the medial meniscus as a secondary stabilizer to anterior tibial translation, particularly during the ACL graft maturation period [24]. Our findings suggest that combined suture techniques may partially mitigate this increased risk, as the relative benefit of combined versus horizontal sutures was greater for medial meniscus repairs.

The protective effect of concurrent ACL reconstruction on meniscal repair healing has been well-established in the literature. Schweizer et al. [7] reported a pooled failure rate of 8.5% for meniscal repairs performed with concomitant ACL reconstruction compared to 14% in ACL-intact knees. This protective effect has been attributed to several factors including restoration of knee stability reducing stress on the repair construct, enhanced biological environment from tunnel drilling and hemarthrosis, and the more rigorous rehabilitation protocols typically employed after combined procedures [25]. Our overall failure rate of 11.8% falls within the expected range for this patient population and compares favorably to historical reports.

Patient-reported outcomes demonstrated clinically meaningful differences among suture configuration groups, with the combined suture group achieving mean IKDC scores of 87.6 compared to 79.8 in the horizontal mattress group. This 7.8-point difference exceeds the minimal clinically important difference (MCID) for the IKDC score, which has been reported as 6.3 to 11.5 points in meniscal surgery populations [26]. Similarly, the combined suture group demonstrated superior return to preinjury sport levels (71.9%) compared to horizontal mattress (51.7%), a clinically important outcome for the typically young and active patients undergoing these procedures.

Objective knee stability assessment using KT-1000 arthrometer revealed significant differences among groups, with the combined suture group demonstrating the lowest side-to-side difference (1.8 mm) and highest proportion of patients with normal stability (< 3 mm difference, 90.6%). While these differences may partly reflect the protective effect of successful meniscal repair on ACL graft function, the magnitude of difference between suture configuration groups was unexpected [27]. The medial meniscus in particular serves as a secondary restraint to anterior tibial translation, and successful repair may provide an important contribution to overall knee stability beyond that provided by ACL reconstruction alone.

MRI assessment of meniscal healing demonstrated significantly higher complete healing rates in the combined suture group (78.1%) compared to horizontal mattress (56.9%). These imaging findings correlated well with clinical outcomes, supporting the validity of MRI as a tool for assessing meniscal repair healing in the postoperative period [28]. The higher rate of incomplete healing in the horizontal mattress group may explain the increased failure rate observed in this group, as partially healed repairs may be more susceptible to secondary tear with return to activity.

Several limitations of this study warrant consideration. First, the retrospective design introduces an inherent potential for selection bias, as suture configuration choice was determined by surgeon preference rather than randomization. Although baseline demographics and clinical characteristics were well-balanced among the three groups, we only partially captured the intraoperative factors that may have influenced construct selection. Tear length, chronicity proxy (injury-to-surgery interval), tear zone, and tear pattern were recorded and incorporated into adjusted analyses, but tissue quality, tear reducibility, and posterior extension were not documented with a standardized scale. These unmeasured factors may have influenced the surgeon’s choice of suture technique and could have contributed to outcome differences. The absence of a standardized allocation protocol means that selection bias cannot be entirely excluded, and the observed associations between suture configuration and outcomes should be interpreted as hypothesis-generating rather than definitive causal relationships. Second, outcomes were assessed through retrospective chart review rather than prospective standardized assessment, which may introduce measurement variability. Third, MRI interpretation of meniscal healing remains imperfect, with studies demonstrating variable correlation between imaging findings and second-look arthroscopic assessment [29]. Fourth, this study did not include a formal cost-effectiveness analysis. Although the combined suture technique required approximately 10 additional minutes of operative time and correspondingly longer tourniquet time, the potential cost savings associated with reduced reoperation rates were not quantified. Future studies incorporating health economic endpoints would be valuable to determine whether the upfront investment in longer operative time is offset by the reduced downstream costs of fewer reoperations and improved functional recovery. Fifth, the minimum 24-month follow-up, while acceptable for evaluating short- to medium-term outcomes and early meniscal repair failure, may not capture the full spectrum of delayed failures. Previous long-term studies have demonstrated that meniscal repair failures can occur beyond 5 years postoperatively, and the true failure rate may therefore be underestimated in the present analysis [29]. Longer-term prospective follow-up extending to a minimum of 5–10 years is strongly recommended to confirm the durability of these findings and to assess the potential impact of different suture configurations on the development and progression of osteoarthritis.

The implications of these findings for clinical practice are significant. Based on our results, combined vertical-horizontal suture configurations should be considered and may be preferred in appropriately selected posterior horn tears repaired concurrently with ACL reconstruction, rather than interpreted as a routine mandate for every case. While this technique requires slightly longer operative time (approximately 10 additional minutes), the meaningful improvements in clinical outcomes and reduction in reoperation rates may justify this investment. This recommendation is particularly important for medial meniscus repairs, where the consequences of failure are more significant [30].

Future research should focus on prospective randomized controlled trials to confirm these findings, optimization of suture density and spacing within combined suture constructs, evaluation of biological augmentation strategies to further enhance healing rates, and longer-term follow-up to assess the durability of meniscal repairs and progression to osteoarthritis [31]. Additionally, comparative effectiveness research examining different all-inside device systems and suture materials would provide valuable guidance for surgical decision-making. The development of validated risk prediction models incorporating suture configuration, tear characteristics, and patient factors could enable personalized treatment recommendations for this common clinical scenario [32].

## Conclusions

This retrospective comparative study demonstrates that combined vertical-horizontal suture configurations for posterior horn meniscal repair in patients undergoing concurrent ACL reconstruction were associated with superior clinical outcomes, improved knee stability, and significantly lower reoperation rates compared to single-plane suture techniques. The horizontal mattress configuration demonstrated the highest failure rate (17.2%), while the combined suture technique achieved the lowest failure rate (7.8%). Medial meniscus location and horizontal suture configuration were identified as independent risk factors for repair failure. These findings support consideration of combined suture configurations as a preferred option in suitable cases, while acknowledging that prospective randomized studies are still needed before causal or routine-use recommendations can be made.

## Data Availability

The datasets generated and/or analyzed during the current study are available from the corresponding author on reasonable request.
